# Rare Taxa Exhibit Disproportionate Cell-Level Metabolic Activity in Enriched Anaerobic Digestion Microbial Communities

**DOI:** 10.1128/mSystems.00208-18

**Published:** 2019-01-22

**Authors:** Yangyang Jia, Marcus H. Y. Leung, Xinzhao Tong, David Wilkins, Patrick K. H. Lee

**Affiliations:** a School of Energy and Environment, City University of Hong Kong, Kowloon, Hong Kong SAR; University of Colorado Denver

**Keywords:** anaerobic digestion, ZOTU, cellulose, population genome, rRNA/rDNA ratio, xylan

## Abstract

Microbial communities are composed of populations with vastly different abundances and levels of metabolic and replicative activity, ranging from actively metabolizing and dividing to dormant or nonviable. The 16S rRNA/rDNA ratio is an emerging tool for evaluating cell-level metabolic activity independent of abundance. In this study, we used five long-term enriched model anaerobic digestion (AD) communities to investigate community composition, diversity, structure, and in particular activity based on the rRNA/rDNA ratio. We cross-validated the 16S amplicon-based results using two alternative operational taxonomic unit (OTU) formation methods (conventional 97% sequence similarity and 100% sequence similar zero-radius OTUs by UNOISE3) and compared these to metagenome-derived population genomes and metatranscriptomes. Significant positive correlations were observed between microbial total activity and abundance with both the amplicon- and omic-based methods. All three methods revealed disproportionately high transcription/abundance ratios for some rare taxa but lower ratios for most abundant taxa for all the communities, which was further corroborated by the high replication rate (iRep) of most low-abundance population genomes.

**IMPORTANCE** Variation in microbial activity levels is increasingly being recognized as both an important dimension in community function and a complicating factor in sequencing-based survey methods. This study extends previous reports that rare taxa may contribute disproportionately to community activity in some natural environments, showing that this may also hold in artificially maintained model communities with well-described inputs, outputs, and biochemical functions. These results demonstrate that assessment of activity levels using the rRNA/rDNA ratio is robust across taxonomic unit formation methods and is independently corroborated by omics methods. The results also provide insight into the comparative advantages and disadvantages of different taxonomic unit formation methods in amplicon sequencing studies, showing that UNOISE3 provides comparable microbial diversity, structure, and activity information as the 97% sequence similarity method but potentially loses some phylogenetic diversity and creates more “phantom taxa” (which are present in the RNA pool but not the corresponding DNA pool).

## Abstract (precis)

Variation in microbial activity levels is increasingly being recognized as both an important dimension in community function and a complicating factor in sequencing-based survey methods. This study extends previous reports that rare taxa may contribute disproportionately to community activity in some natural environments, showing that this may also hold in artificially maintained model communities with well-described inputs, outputs, and biochemical functions. These results demonstrate that assessment of activity levels using the rRNA/rDNA ratio is robust across taxonomic unit formation methods and is independently corroborated by omics methods. The results also provide insight into the comparative advantages and disadvantages of different taxonomic unit formation methods in amplicon sequencing studies, showing that UNOISE3 provides comparable microbial diversity, structure, and activity information as the 97% sequence similarity method but potentially loses some phylogenetic diversity and creates more “phantom taxa” (which are present in the RNA pool but not the corresponding DNA pool).

## INTRODUCTION

Bioconversion of carbon and other nutrients in wastewater via anaerobic digestion (AD) is achieved by a community of microbes (1). The composition, diversity, structure, and metabolic activity of these microbial communities all affect how well anaerobic digesters perform (2). Many previous studies have investigated the composition, diversity, and structure of AD microbial communities by evaluating the 16S rRNA gene (rDNA) (3–5), while others have assessed potential metabolic capabilities by examining microbial metagenomes (6–8). However, DNA-based microbial community surveys can provide information only on the total community and are unable to discriminate between microbes with different levels of metabolic activity. While significant differences have been demonstrated between the total and active microbial communities in full-scale anaerobic digesters based on 16S rDNA/rRNA amplicon data (9), the activity levels of participating microbes have not been examined in detail. Microbes can be growing (undergoing cell division), active (performing metabolic functions but not dividing), dormant (neither dividing nor metabolizing), or recently deceased, and thus, they participate in ecological functions to different degrees (10).

As the number of 16S rRNA transcripts in a cell is positively correlated with its metabolic activity and/or growth rate (11–13), the metabolic state of a population of cells could be inferred by quantitative rRNA sequencing (14). The ratio between transcribed and genomic 16S rRNA sequences (rRNA/rDNA ratio) is a means of normalizing rRNA transcription against cell count, and it has been used to compare metabolic activity between populations (15, 16). The rRNA/rDNA ratio has been used to study the active microbial communities in aquatic (17–20), ice sheet (21), air (22, 23), soil (24–27), and activated-sludge (28, 29) environments, but it has not yet been widely applied in anaerobic digesters (30). Furthermore, as microbial ribosomal amplification is highly variable across both taxonomy and ecological strategy, the rRNA/rDNA ratio may not always be sufficient to discriminate between microbes in different metabolic states (10), and it may be particularly prone to error in populations with a mixture of amplification levels (31).

In previous studies, the rRNA/rDNA ratio has been calculated at the level of operational taxonomic units (OTUs), conventionally represented as clusters of 16S rRNA gene sequences with at least 97% sequence similarity. However, a growing number of recent studies suggest that the 97% sequence similarity threshold does not necessarily capture phylogenetically and ecologically homogeneous microbial populations (32, 33), and it may underestimate species richness by grouping dissimilar taxa. This has led to the proposal of studying taxonomic units at a single-nucleotide resolution, and they are called “zero-radius OTUs” (ZOTUs), exact sequence variants (ESVs), amplicon sequence variants (ASVs), features, or sub-OTUs (34–36). Because the formation of such units is exquisitely sensitive to sequencing error, 100% sequence similarity-based methods rely on denoising and error correction algorithmic approaches to infer accurate ESVs from potentially noisy sequencing data (35–37). A number of packages (e.g., DADA2, Deblur, and UNOISE3), each with its own strengths as compared previously (38), are available to generate ESVs. These methods are best conceptualized as alternative methods of forming taxonomic units rather than differing from conventional OTU formation merely by the choice of the sequence similarity threshold. For example, UNOISE3 is an algorithm that depends on the frequency of occurrence of a read, but not the sequencing quality scores, and two parameters with preset values to infer correct biological sequences from the erroneous ones (35). It is not currently known how well different taxonomic unit formation methods accurately capture the composition, diversity, and structure of AD communities, nor how well rRNA/rDNA ratios based on 100% sequence similar taxonomic units reflect their true metabolic activity.

We have previously established five microbial AD communities capable of digesting cellulose or xylan to CH_4_ at mesophilic (35°C) or thermophilic (55°C) conditions (39) and reconstructed 107 population genomes and their transcriptional activity from combined metagenomic and metatranscriptomic sequencing of these communities (40). Because these reconstructed population genomes are not sensitive to sequence similarity thresholds or minor sequencing errors, and the transcription of structural genes is not directly dependent on variance in ribosomal amplification, these population genomes and their transcription profiles provide another means of revealing microbial metabolic activity, so can be used to validate the use of alternative taxonomic units and rRNA/rDNA ratios to assess AD communities.

In this study, we sequenced 16S rDNA and rRNA amplicons from our five enriched AD microbial communities and assessed microbial community structure and activity using rRNA/rDNA ratios with both the conventional OTU (97% sequence similarity) and ZOTU (single-nucleotide resolution taxonomic unit by UNOISE3 [35]) methods. We also used metagenomic and metatranscriptomic (mRNA) sequencing data from the same samples to calculate transcription/abundance ratios for reconstructed population genomes, allowing us to cross-validate and identify relative biases in the 16S amplicon-based methods.

## RESULTS AND DISCUSSION

### Overview of total and active communities.

AD of cellulose or xylan to CH_4_ is achieved by a diverse microbial community performing a range of functions at different levels of metabolic activity. In this study, partial 16S rDNA and 16S rRNA regions were sequenced to query the total and active microbial communities, and taxonomic units formed with either conventional 97% sequence similarity clustering (OTUs) or denoising with UNOISE3 at 100% similarity (ZOTUs). With both methods, the rDNA (total community) and rRNA (active community) reads captured largely identical sets of taxonomic units (see Table S1 in the supplemental material). From each sample, 83 to 268 OTUs and 86 to 263 ZOTUs were identified in both the rDNA and rRNA read sets, accounting for >98% of the rDNA and >98% of the rRNA reads in all but one sample (SWH-C-D15). While all samples yielded some taxonomic units that were identified in only the rDNA or rRNA read sets, these represented <2% of the total reads for either read set, again with the exception of SWH-C-D15. These read set-specific taxonomic units ranged in number from 11 to 117 (OTUs) and 13 to 141 (ZOTUs) per sample. In SWH-C-D15, a high proportion (35 to 36%) of rRNA reads contributed to OTUs or ZOTUs that were not identified in the rDNA read set.

10.1128/mSystems.00208-18.6 TABLE S1 Summary of the proportion of overlaps between 16S rDNA and 16S rRNA reads in each sample based on OTU clustering and ZOTU denoising methods. Download Table S1, XLSX file, 0.01 MB. Copyright © 2019 Jia et al. 2019 Jia et al. This content is distributed under the terms of the Creative Commons Attribution 4.0 International license.

Taxonomic units that were detected exclusively in the rDNA read set might represent microbes that were present but not metabolically active (i.e., dormant or dead). Conversely, taxonomic units exclusively detected in the rRNA read set represent “phantom taxa,” where the rDNA template was not successfully identified due to either undersampling of rDNA or the introduction of nucleotide errors during reverse transcription (an error rate of ∼1/15,000 for reverse transcriptase) (41), PCR (an error rate of ∼1/10,000 to 1/50,000 for *Taq* polymerase [41]), or sequencing (22). rDNA undersampling may be more likely in the case of rare taxa which are nonetheless highly active and/or have high ribosomal amplification, which would yield abundant rRNA transcripts relative to scarce rDNA genes. Either of these explanations is consistent with the higher proportion of phantom taxa among ZOTUs (31% of ZOTUs) compared to OTUs (11%). A single-nucleotide error introduced in PCR or sequencing and not corrected by denoising will either create a phantom ZOTU or cause the sequence to be misclassified into an incorrect ZOTU, where the same error may have no effect on OTUs formed by 97% similarity clustering. Similarly, a single rRNA read from a taxon that did not yield an rDNA read due to undersampling might not create a phantom taxon if the read was included in a 97% similar OTU for which at least one complementary rDNA sequence was detected, but such concealment is not afforded by 100% similar ZOTUs.

### Community diversity and structure.

While 80% of filtered reads were successfully clustered into OTUs, only 48% were assigned to ZOTUs. Despite this, OTU and ZOTU richness across all samples did not differ significantly among either the total (rDNA) or active (rRNA) communities (Table 1). This suggests that the tendency of the 100% similarity threshold to “split” taxa that would otherwise be “lumped” at the 97% threshold mostly compensated for the decreased number of reads retained in the ZOTU set, although it does not indicate which of the two methods achieved a more accurate estimate of the ecologically meaningful taxon richness. Faith’s phylogenetic diversity (PD) index was significantly higher among OTUs compared to ZOTUs in both the total and active communities, suggesting that the OTU method was able to capture a greater degree of phylogenetic diversity than the ZOTU method, likely because of the large number of low-abundance reads discarded during ZOTU formation. The Shannon index of diversity was significantly higher among ZOTUs compared to OTUs in both the total and active communities, but as the two taxonomic unit formation methods yielded similar richness, this is likely due to greater abundance evenness among ZOTUs, which again may be attributable to the discarding of low-abundance reads.

**TABLE 1:** Mean values across all samples of alpha-diversity indices in the 16S rDNA and 16S rRNA read sets for the OTU and ZOTU taxonomic formation methods^*a*^

Alpha-diversity index	Read set	Mean (SD) value for diversity index by the following taxonomic formation method:		*P*^*b*^
		OTU	ZOTU	
Richness	rDNA	240 (68)	240 (56)	0.67
	rRNA	240 (72)	220 (47)	0.20
Faith’s PD	rDNA	7.9 (1.6)	5.0 (0.82)	0.00*
	rRNA	8.2 (2.1)	5.3 (1.2)	0.00*
Shannon index	rDNA	3.4 (0.7)	4.2 (0.6)	0.00*
	rRNA	3.7 (0.8)	4.4 (0.64)	0.02*

a Alpha-diversity was calculated after all samples were normalized to a read depth of 31,560 or 17,707 for OTUs and ZOTUs, respectively, by randomly subsampling 10 times, and values were averaged.

b *P* values are for Mann-Whitney tests for a significant difference between methods for the given index and read set. *P* values of <0.05 are indicated by an asterisk.

At the rank of family, the 16S rDNA (total community) and 16S rRNA (active community) sample taxonomic compositions were broadly similar for both the OTU and ZOTU taxonomic unit formation methods (Fig. S1). The exception was SWH-C-D15, which was generally consistent in taxonomic composition between methods, but had a large population of *Mycoplasmataceae* and *Rhizobiaceae* taxa in the rRNA community which were not identified in the rDNA community. These represent phantom taxa, which as described above were unusually abundant in SWH-C-D15. Members of the *Mycoplasmataceae* and *Rhizobiaceae* families are not known to be prevalent in AD communities, and these sequences could be contaminants (they were present in one of the five negative controls at a relative abundance of <6.8%). The amplicon-based taxonomic compositions were also reflected in our previously reported population genomes (40), of which the bacterial families *Clostridiaceae, Ruminococcaceae*, and *Veillonellaceae* and the methanogenic archaeal family *Methanobacteriaceae* were among the most abundant taxa in most samples.

10.1128/mSystems.00208-18.1 FIG S1 Taxonomic composition of the total and active communities in the enrichment culture samples at the taxonomic rank of family based on OTU clustering and ZOTU denoising results. The top 12 families across all samples were plotted, with all the others grouped into the “Minor/Unclassified” category. Many sequences could not be annotated at the genus level. Download FIG S1, TIF file, 1.4 MB. Copyright © 2019 Jia et al. 2019 Jia et al. This content is distributed under the terms of the Creative Commons Attribution 4.0 International license.

**FIG 1:**
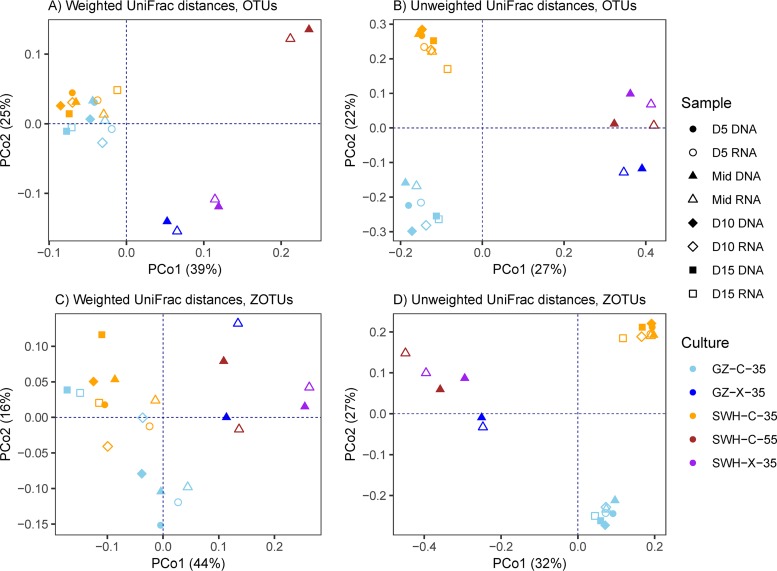
Principal-coordinate analysis of the pooled total (rDNA) and active (rRNA) communities as measured with the weighted (A and C) and unweighted (B and D) UniFrac distances, using either the OTU (A and B) or ZOTU (C and D) taxonomic unit formation method. The percentage in parentheses on each axis gives the estimated contribution of each principal coordinate to the total variance.

We have previously reported that the 16S rDNA profiles of these enrichment cultures differ significantly by enrichment condition (39). This was found to hold true for both read clustering and denoising methods using profiles that combined rDNA and rRNA OTUs/ZOTUs (Fig. 1), with significant differences between enrichment conditions based on both weighted (PERMANOVA pseudo-F = 12.19, *P* = 0.001 for OTU; pseudo-F = 7.504, *P* = 0.001 for ZOTU) and unweighted (PERMANOVA pseudo-F = 8.823, *P* = 0.001; pseudo-F = 15.92, *P* = 0.001 for ZOTU) UniFrac distances (all time points pooled for each enrichment condition).

### Community-level correlations between taxon abundance and transcriptional activity.

Previous 16S amplicon sequencing studies have demonstrated that the relative abundance (contribution to total rDNA) and relative metabolic activity (contribution to total rRNA) of microbial taxa are significantly and positively correlated in a range of environments, including coastal ocean (20), benthic glacier streams (17), and soil (26). This is also a key premise of the rRNA/rDNA method of inferring cell-level microbial activity, as it is assumed that after rRNA abundance is normalized against rDNA abundance, any remaining variation can be attributed to cell-level differences in ribosomal amplification.

**FIG 2:**
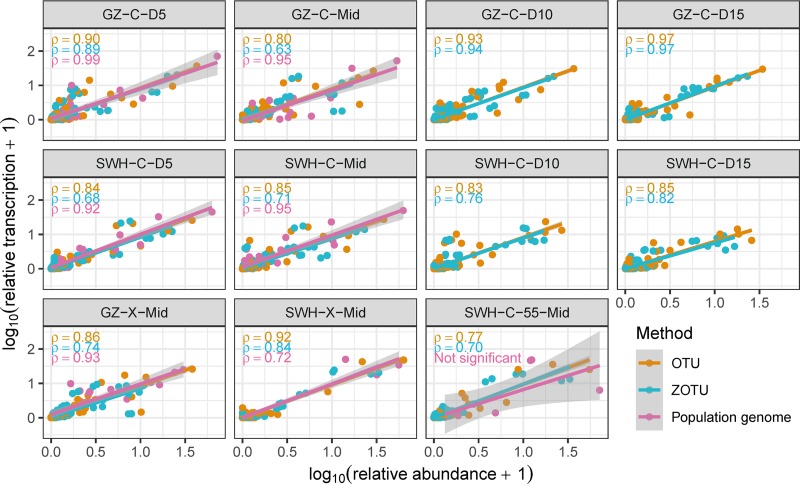
Relationship between relative abundance and relative transcription (logarithmic scales) of OTUs (brown), ZOTUs (turquoise), and population genomes (pink) across all samples. Each symbol represents one taxonomic unit. Lines represent linear models fitted for each set of taxonomic units in each sample. For OTUs and ZOTUs, abundance and transcription are the relative rDNA and rRNA abundances, respectively; for population genomes, they are derived from metagenomic and metatranscriptomic abundances. Pearson’s ρ (when *P* < 0.05) for each set of taxonomic units is given in the top left-hand corner of each plot.

We found a positive and significant linear correlation between rRNA and rDNA relative abundances across both clustering and denoising methods and all samples (Fig. 2; Pearson’s ρ ≥ 0.63, *P* < 0.0001 for all samples). There was no significant difference between the correlation coefficients obtained using the OTU clustering versus ZOTU denoising methods (Mann-Whitney *P* = 0.08). For the samples from which population genomes were reconstructed from metagenomes, we also tested for a linear correlation between population genomes’ metagenomic and metatranscriptomic relative abundances. In all but one sample (SWH-C-55-Mid), a significant positive linear correlation was found (Fig. 2; Pearson’s ρ ≥ 0.72, *P* < 0.001 for all samples except SWH-C-55-Mid). On average, the 10 most abundant taxa in each sample contributed 79% (OTUs), 69% (ZOTUs), or 95% (population genomes) of RNA production (Fig. S2).

10.1128/mSystems.00208-18.2 FIG S2 Cumulative relative production compared to the rank abundance of OTUs (brown), ZOTUs (turquoise), and population genomes (pink) for each culture. Each symbol represents one taxonomic unit. For OTUs and ZOTUs, production of a taxon is its relative contribution to total rRNA; for population genomes, it is derived from the contribution of transcripts from a population genome to total mRNA. Download FIG S2, TIF file, 2.6 MB. Copyright © 2019 Jia et al. 2019 Jia et al. This content is distributed under the terms of the Creative Commons Attribution 4.0 International license.

The positive relationship between genomic and transcriptional abundance and the rapid accumulation of cell production support previous reports that the most abundant community members are also the greatest contributors to total community metabolic activity, independent of variation in cell-level metabolic activity between taxa (42). This is unlikely to be an artifact of “lumping” dissimilar taxa during the clustering or denoising of amplicon-based reads (32), as the correlation was consistent between the OTU and ZOTU methods and was corroborated by the reconstructed population genomes, which are not susceptible to clustering artifacts.

### Cell-level metabolic activity.

The rRNA/rDNA ratio of a taxonomic unit has been used to infer its cell-level metabolic activity or growth rate, normalized against its population abundance. We found that taxonomic units formed using the OTU method had slightly higher average rRNA/rDNA ratios (mean = 1.5, SD = 8.1) than ZOTUs (mean = 1.4, SD = 5.1), though the difference was not statistically significant (Mann-Whitney *P* = 0.83). The rRNA/rDNA ratio ranged from 0 to 316 for OTUs and from 0 to 206 for ZOTUs, with approximately 50% of both sets of taxonomic units having a ratio smaller than one across all samples. In contrast, the transcription/abundance ratios of reconstructed population genomes had a similar mean (1.5) but much lower variance (SD = 2.9; range, 0 to 26). As the population genome transcription levels were estimated from the transcription of ORFs in the population genome scaffolds, this difference in variance may reflect a higher variance in ribosomal amplification between taxa relative to variance in the transcription level of structural genes.

**FIG 3:**
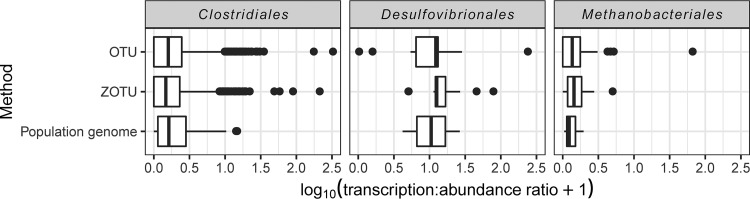
Transcription/abundance ratios (logarithmic scale) of OTUs, ZOTUs, and population genomes of selected microbial orders. For OTUs and ZOTUs, the transcription/abundance ratio is the rRNA/rDNA ratio; for population genomes, it is derived from metagenomic and metatransciptomic abundances. The box indicates the 25th to 75th percentiles, the thick vertical line indicates the median, and whiskers represent smallest and largest values no more than 1.5× interquartile range.

To compare the inferred metabolic activities of 16S amplicon-based taxonomic units against those of the population genomes, we examined three high-abundance orders for which a number of representative population genomes had been reconstructed (Fig. 3). The *Clostridiales* had per-sample rRNA/rDNA ratios ranging from 0 to 316 among OTUs and from 0 to 206 among ZOTUs and transcription/abundance ratios from 0 to 13.4 among population genomes but had similar mean ratios with all three methods (OTU mean = 1.4, ZOTU mean = 1.3, population genome mean = 1.6), although the ratios were found to differ significantly between methods using an analysis of variance (Kruskal-Wallis *P* = 0.001).

The *Methanobacteriales* had similarly low ratios across all three methods (OTU range = 0 to 64, mean = 0.93; ZOTU range = 0 to 3.9, mean = 0.58; population genome range = 0.05 to 0.96, mean = 0.34), with no statistically significant differences between methods (Kruskal-Wallis *P* = 0.20). While this consistently low ratio might suggest that the *Methanobacteriales* are mostly dormant, we note that methane production was demonstrated for all the mesophilic enrichment cultures (39, 40), suggesting that the *Methanobacteriales* were in fact metabolically active. This apparent contradiction may be due to the fact that ribosomal amplification is not always linearly correlated with metabolic activity (10), and it could also reflect the tendency for mixed-growth rate communities and undersampling to falsely suggest that active taxa are dormant (31).

rRNA/rDNA and transcription/abundance ratios for the order *Desulfovibrionales* (of which only the genus *Desulfovibrio* was detected using either the amplicon-based or population genome methods) tended to be higher than that for the other two orders, suggesting a higher level of metabolic activity. The ratios ranged from 0 to 232 (mean = 32) among OTUs, 4.0 to 76 (mean = 18.4) among ZOTUs, and 3.2 to 26 (mean = 14.3) among population genomes, with no statistically significant difference between the three methods (Kruskal-Wallis *P* = 0.68). Despite this high transcriptional activity, the order was not abundant, with an average relative abundance of <0.3% (0 to 1.1% of OTUs and 0 to 0.7% of ZOTUs) among the amplicon-based populations. While high rRNA/rDNA ratios were detected from *Desulfovibrionales* in all the mesophilic cellulose culture samples, no *Desulfovibrionales* were detected in the mesophilic xylan or thermophilic cellulose cultures. Only one *Desulfovibrionales* population genome was reconstructed from a metagenome, and it was found only in the SWH-C-35 culture at two time points, where it exhibited transcription/abundance ratios of 3.2 and 26.

Variation in neither ribosomal amplification (10) nor the transcription of structural genes is necessarily directly related to growth or metabolic activity, and different growth strategies may entail different levels of transcriptional regulation. For example, the oligotrophic marine bacterium “*Candidatus* Pelagibacter ubique” (SAR11), which has an atypically small and streamlined genome, has been reported not to vary the transcription of >99% of its genes in response to growth rate and to transcribe 30S and 50S ribosomal genes at much lower levels than taxa with a copiotrophic growth strategy (43). However, the concordance between amplicon rRNA/rDNA and population genome transcription/abundance ratios suggests that both methods capture at least some of the true variance in metabolic and growth rates between taxa.

**FIG 4:**
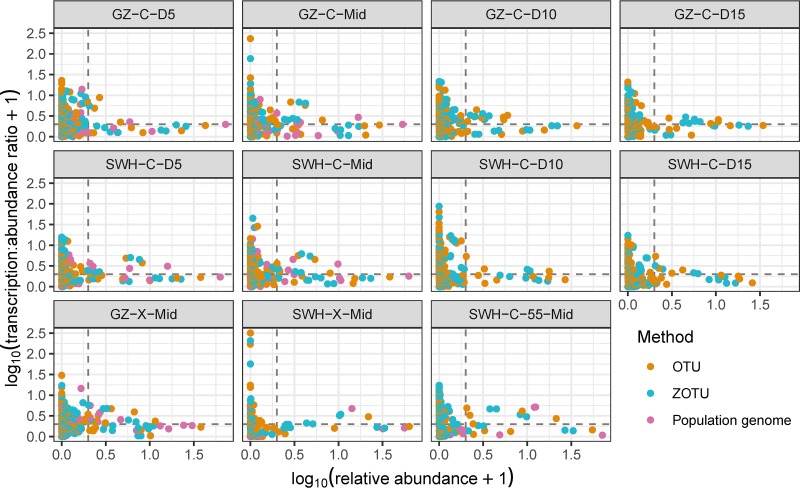
Relationship between relative abundance and transcription/abundance ratio (logarithmic scales) of OTUs (brown), ZOTUs (turquoise), and population genomes (pink) across all samples. Each symbol represents one taxonomic unit. For OTUs and ZOTUs, abundance and transcription are the relative rDNA and rRNA abundances, respectively; for population genomes, they are derived from metagenomic and metatranscriptomic abundances. The gray dashed lines indicate a relative abundance of 1% (vertical line) and a transcription/abundance ratio of 1 (horizontal line).

We compared the transcription/abundance ratios of 16S amplicon taxonomic units and population genomes against their relative abundances (Fig. 4). For all three methods, a pattern emerged in which taxonomic units with the highest rRNA/rDNA or transcription/abundance ratios were almost always those with low relative abundances, with the majority of taxonomic units with ratios of >1 having relative abundances of <1%. Other studies have reported a similar pattern in marine environments (18–20), as well as in outdoor air (22) and indoor air (23), and activated-sludge systems (28), although as noted above and by others (42), despite this pattern, the vast majority of total community production is still contributed by high-abundance taxa (Fig. S2).

There are several possible explanations for this observation. It may arise from methodological artifacts, such as PCR bias or undersampling. Steven et al. (31) reported that undersampling of simulated microbial communities can increase the apparent range of rRNA/rDNA ratios, presumably due to the scarcity of rDNA relative to rRNA. The higher number of rare compared to abundant taxonomic units could also give rise to a higher absolute number of extreme rRNA/rDNA ratios by random chance, even if variance in ratios was identical between rare and abundant taxa. Alternatively, this may be a true biological pattern arising from the microbial community dynamics and/or structure. The “kill-the-winner” hypothesis suggests that fast-growing taxa may be more prone to lysis and grazing, preventing them from being dominant, while the slow-growing taxa are more resistant and thus can flourish (44). This hypothesis has been used to explain the higher growth rate of the rare taxa and the lower growth rate of the abundant taxa in marine environments (20, 42).

**FIG 5:**
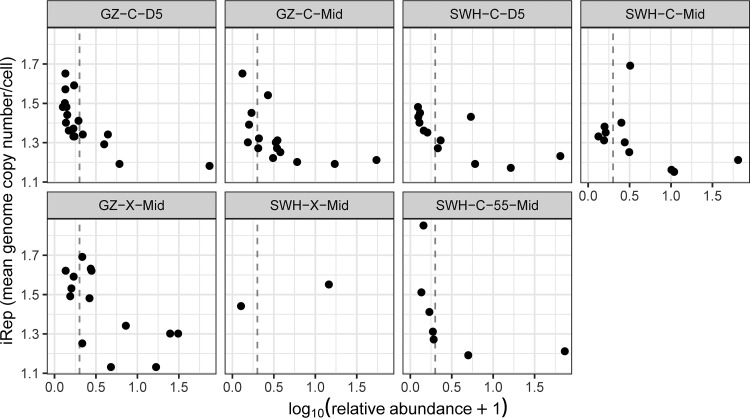
iRep index (estimated genome copy number/cell) compared to relative abundance (logarithmic scale) for population genomes. Each symbol represents a population genome. The vertical gray dashed lines indicate a relative abundance of 1%.

To further investigate whether this pattern was biological or artifactual, we estimated the replication rates of the population genomes using the iRep metric, which is derived from variable read coverage across the length of a genome (45), and thus provides corroboration of microbial activity independent of the transcription/abundance ratios. We found a similar pattern of high-iRep genomes being almost exclusively low abundance (Fig. 5), although the linear correlation between the iRep metric and the transcription/abundance ratio was only weakly positive (Pearson’s ρ = 0.22, *P* = 0.048). Taken together, this suggests that while methodological artifacts may influence observed rRNA/rDNA ratios, the rRNA/rDNA method can capture biologically meaningful patterns using either OTU or ZOTU taxonomic unit formation method even in the presence of undersampling.

Close examination of the transcriptional profiles of the top three genomes with a high transcription/abundance ratio but low relative abundance (<1%) in each sample revealed that the top 20 transcribed ORFs spanned a wide range of biological functions, although most of the proteins could not be classified based on the SEED system category (46, 47) (Fig. S3). Among the most highly transcribed ORFs, they encode a membrane transporter protein, a rubrerythrin and a zinc finger domain protein (Fig. S3). These proteins are known to perform substrate uptake, stress protection, or diverse molecular recognition and binding functions. Hence, microbial populations that are rare but have a high transcription/abundance ratio could play important functional roles in AD communities.

10.1128/mSystems.00208-18.3 FIG S3 Biological functional category and transcription level of the top 20 transcribed ORFs in the top three genomes with a high transcription/abundance ratio and low relative abundance (<1%) in each sample. (A) The number of the ORFs in each SEED system category. (B) Transcription level of the ORFs represented as TPM in each SEED system category. ORFs with a TPM greater than 2,000 were indicated with an asterisk together with functions based on the SEED functional role where available. Download FIG S3, TIF file, 1.2 MB. Copyright © 2019 Jia et al. 2019 Jia et al. This content is distributed under the terms of the Creative Commons Attribution 4.0 International license.

### Transcriptional activity dynamics of abundant ZOTUs.

In order to understand to what extent the 97% sequence similarity threshold may cause ecologically different microbes to be “lumped” into the same taxonomic unit, we mapped each ZOTU against the representative sequence for each OTU sequence using a similarity threshold of 97%. A total of 1,318 ZOTUs (87%) were successfully mapped to 425 OTUs, with each OTU recruiting 1 to 29 ZOTUs. The mapping of the majority of ZOTU sequences to a small subset of OTUs is consistent with the lower phylogenetic diversity of the ZOTU-based communities (Table 1).

**FIG 6:**
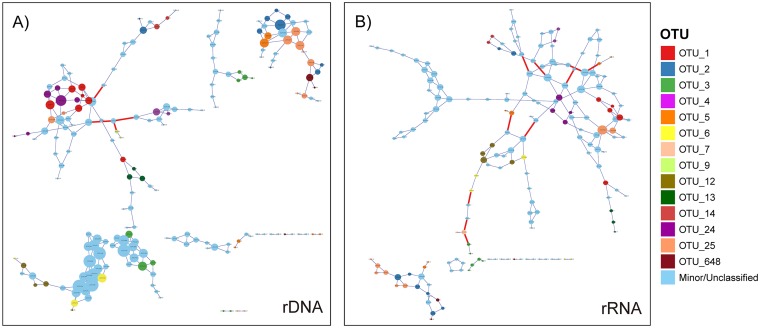
Cooccurrence of abundant ZOTUs determined by the SPIEC-EASI algorithm, in the total (rDNA) (A) and active (rRNA) (B) communities. Nodes indicate ZOTUs, colored by the OTU to which they had ≥97% sequence similarity (ZOTUs not belonging to the 14 most abundant OTUs grouped in “Minor/Unclassified”), with the size of the node proportional to the ZOTU magnitude of correlations with other ZOTUs. Edges indicate significant correlations between ZOTUs, colored by a positive (blue) or negative (red) interaction.

ZOTUs with a 16S rDNA relative abundance greater than 1% in any sample were selected for further analysis. The relative abundances of these ZOTUs ranged from 0 to 32% in each sample, while their rRNA/rDNA ratios ranged from 0 to 15.9. In the two mesophilic cellulose cultures for which time course data were available, ZOTUs mapped to the same OTU showed similar dynamic trends in rDNA relative abundance, rRNA relative abundance, and rRNA/rDNA ratio during the course of fermentation (Fig. S4). Because compositional microbial abundance data are not independent and can exhibit spurious cooccurrence patterns, we used a compositionally robust method (48, 49) to investigate whether ZOTUs mapped to the same OTU tended to have different cooccurrence patterns, which would suggest the inclusion of ecologically distinct taxa within the same OTU. The resulting networks (Fig. 6) tended to closely group ZOTUs from the same OTU into positively interacting clusters in both the total (rDNA) and active (rRNA) communities. No significant negative interactions were observed in the networks between ZOTUs from the same OTU. Overall, the results suggest that, at least among abundant ZOTUs, OTUs tended to cluster together ZOTUs with similar dynamics in abundance and metabolic activity.

10.1128/mSystems.00208-18.4 FIG S4 Dynamic trends in ZOTU rDNA relative abundance, rRNA relative abundance, and rRNA/rDNA ratio in the two cultures for which time series data were collected, GZ-C-35 (A) and SWH-C-35 (B). ZOTUs with abundance of ≥1% in at least one sample are shown. OTUs indicate the OTU to which each ZOTU had a ≥97% sequence similarity match. The horizontal axis indicates day of fermentation time course (D5, day 5; Mid, mid-exponential point; D10, day 10; D15, day 15). Download FIG S4, TIF file, 2.5 MB. Copyright © 2019 Jia et al. 2019 Jia et al. This content is distributed under the terms of the Creative Commons Attribution 4.0 International license.

### Comparison of the ZOTU and conventional OTU methods in evaluating activity.

The use of ESVs, features, or ZOTUs in microbial community surveys has recently been proposed as superior to conventional OTUs formed at 97% sequence similarity, as these new methods may maximize the phylogenetic resolution of the sequencing data while minimizing the conflation of biologically distinct populations into the same taxonomic unit (32, 35, 37). We found that, at least for the UNOISE3 algorithm with default settings and for the types of communities studied (which were simplified following long-term enrichment), there was no clear advantage of the ZOTU method over conventional OTU formation method and that the ZOTU method may indeed discard some biologically relevant information. Both methods provided comparable pictures of the community taxonomic compositions (Fig. S1), though the ZOTU method appeared to capture less phylogenetic diversity (Table 1), and both methods largely agreed with the results of a separate metagenome-based survey in the assessment of relative metabolic activity (40). Both methods, and the reconstructed population metagenomes, appeared susceptible to the type of false-negative error described previously (31), in which taxa confirmed to be active by *in vitro* measurements appeared dormant based on low rRNA/rDNA ratios (Fig. 3). However, the ZOTU method produced a much larger proportion (31%) of phantom taxa than the OTU method (11%). Because phantom taxa are necessarily the result of undersampling and/or methodological error and because these taxa must be excluded from any analysis that relies on relative rDNA and rRNA abundances, this suggests that the ZOTU method may be inferior to conventional OTUs when attempting to assess metabolic activity inferred by the rRNA/rDNA ratio, especially for studies with insufficient sequencing effort or communities with a large number of rare taxa. UNOISE3 uses the abundance of reads to denoise sequences, rather than quality scores and a model of sequencing error as in other methods (37), and it excludes sequences that do not meet an abundance threshold (by default 8) in the pooled input reads (35). Given the large proportion of reads excluded in the ZOTU pipeline compared to the OTU pipeline, it is likely that this default threshold had a significant effect on ZOTU formation and may have resulted in the ZOTU set discarding many genuine low-abundance taxa. We found little evidence to support the proposal that 97% similar OTUs frequently conflate taxa with different ecological roles in our enriched model communities, at least among higher-abundance ZOTUs. Further work is needed to explore whether this holds true for other artificial and natural systems and whether other ZOTU formation algorithms give similar results.

### Conclusions.

Variation in microbial activity levels is an important factor in community function. In our model AD communities, the rRNA/rDNA ratio revealed large variance in cell-level microbial activity levels across taxa, cooperated with two alternative taxonomic unit formation methods and by reconstructed metagenomic population genomes and their complementary transcriptomes. Confirming previous findings, the most metabolically active and rapidly dividing taxa tended to be the least abundant in the five AD communities, though further work is needed to establish whether this is an entirely natural or at least partially artifactual pattern. We found no obvious evidence to support the suggestion that the conventional 97% sequence similarity 16S OTUs conflates distinct ecological entities or discards biologically relevant information compared to the UNOISE3 method applied in this study, and instead found the UNOISE3-based ZOTU method generated a higher rate of phantom taxa, suggesting it may less suitable for assessing metabolic activity with the rRNA/rDNA ratio, a method already fraught with difficulties in interpretation.

## MATERIALS AND METHODS

### Establishment of AD communities.

Five stable anaerobic cultures digesting either cellulose or xylan as the sole carbon and energy source were established after 2 years of enrichment (39), using inocula from two full-scale anaerobic digesters: SWH, an municipal wastewater anaerobic digester in Shek Wu Hui, Hong Kong SAR; and GZ, treating high-strength wastewater from a beverage factory in Guangzhou, China (50). To assess the short-term microbial community dynamics and activity during batch fermentation, replicate batch experiments were set up for the five enrichment cultures as described previously (40). Briefly, time course samples were collected from the two mesophilic cellulose cultures at day 5, the mid-exponential point (between days 5 and 10), day 10, and day 15, and samples at the mid-exponential point were collected from the two mesophilic xylan cultures and the thermophilic cellulose culture. The mid-exponential point was determined from the physiochemical profiles of the cultures using analytical methods as previously described (39). Samples are named following the scheme of “inoculum-substrate-(temperature for 55°C culture only)-time,” where for substrate “C” denotes cellulose, while “X” denotes xylan. For example, sample GZ-C-D5 denotes a sample collected on day 5 from the enrichment culture inoculated from the GZ digester and cultured with cellulose as the sole substrate. All enrichment cultures were incubated at 35°C (mesophilic), except for SWH-C-55, which was incubated at 55°C (thermophilic).

### Nucleic acid extraction and sequencing.

From each of the two biological replicates of each culture at each time point, one 12-ml sample was collected for DNA extraction and one for RNA extraction. DNA extraction (39) and total RNA extraction, removal of genomic DNA, RNA quality assessment, and complementary DNA (cDNA) synthesis (40, 51) were carried out as described previously. Briefly, DNA was extracted using the PowerSoil DNA isolation kit (MO BIO Laboratories, USA) with minor modifications, while total RNA was extracted using the RNeasy minikit (Qiagen, USA) following the manufacturer’s protocol. The quality of total RNA was verified by a Nano Drop 2000 spectrophotometer (Thermo Fisher Scientific, USA) and a Bioanalyzer 2100 (Agilent, USA), contaminating genomic DNA was removed by on-column and in-solution DNase digestion with an RNase-free DNase set (Qiagen, USA), and reverse transcription into cDNA was performed using the Superscript III (Invitrogen, USA) reverse transcriptase following the manufacturer’s instructions. Nucleic acid samples extracted from biological replicates were pooled by equal mass for downstream processing. PCR targeting the 16S V4 hypervariable region was performed using the 515F/806R primer pair (52). Triplicate reactions were performed for each sample, which were then pooled and purified before indexing-PCR. Libraries were prepared using the Illumina MiSeq reagent kit v2 and sequenced on the MiSeq platform by Health GeneTech Corporation (Taoyuan City, Taiwan), generating ∼250-bp paired-end reads. Only forward reads were used for further analysis. A total of 22 rDNA and rRNA samples were sequenced, as well as five blank samples (empty extraction kit tube) as negative controls. The metagenomics (40) and 16S rDNA (this study) or metatranscriptomics (40) and 16S rRNA (this study) sequences were generated from the same DNA or total RNA samples, respectively.

### Read quality control and taxonomic unit formation.

16S rDNA and rRNA reads were processed in parallel. The “fastq_filter” command in USEARCH (53) was used to trim and filter raw sequence reads using the criteria of minimum read length of 230 bp and a maximum of one expected error per read, retaining a total of 1,520,585 high-quality reads (64% of raw reads). Trimmed and filtered sequencing reads were used to form OTUs and ZOTUs using the “-cluster_otus” (54) and “-unoise3” (35) commands in USEARCH, respectively. Singleton OTUs or ZOTUs with fewer than eight reads were removed using the default “-minsize” values. The “-usearch_global” command in USEARCH was used to map the filtered high-quality reads to OTUs and ZOTUs at minimum sequence identities of 97% and 100%, respectively. The “-usearch_global” command with a minimum sequence identity of 97% was also used to map ZOTU sequences to OTU sequences. Chimeric OTUs/ZOTUs were removed using the “uchime_ref” command in USEARCH. OTUs/ZOTUs represented by >5% of reads on average in the negative controls were deemed likely contaminants and were removed from all samples.

Following quality control, a total of 1,158 OTUs and 1,221 ZOTUs were retained, accounting for 1,222,807 (31,560 to 66,169 per sample) and 736,782 (17,707 to 41,275 per sample) reads, respectively (see Table S2 in the supplemental material). Taxonomic information was assigned to each OTU/ZOTU with the “assign_taxonomy.py” script from QIIME (54) with default settings, using the Greengenes 16S rRNA gene sequence database (version 13_8).

10.1128/mSystems.00208-18.5 TABLE S2 Sample description, sequencing summary, and the rarefied alpha-diversity for all samples. Download Table S2, XLSX file, 0.01 MB. Copyright © 2019 Jia et al. 2019 Jia et al. This content is distributed under the terms of the Creative Commons Attribution 4.0 International license.

### Analysis of diversity, structure, and metabolic activity.

Taxonomic unit richness, Faith’s phylogenetic diversity (FPD), and the Shannon diversity index were calculated using the “alpha_diversity.py” command in QIIME after all the rDNA and rRNA samples were normalized to 31,560 or 17,707 reads for OTUs and ZOTUs, respectively, by randomly subsampling to the normalization count 10 times and averaging the results. The weighted and unweighted UniFrac distances (55) between samples were calculated using the “beta_diversity.py” command in QIIME. Principal-coordinate analysis (PCoA) ordination of the UniFrac distances between samples was performed with the R package vegan (2.4-0). PERMANOVA pseudo-F statistics for significant differences between culture conditions based on UniFrac distances were calculated using QIIME with 999 permutations. Sparse inverse covariance estimation for ecological association inference (SPIEC-EASI) (48, 49) was used to assess potential ecological interactions between ZOTUs for the total and active microbial communities, respectively. Cytoscape (version 3.5.1) was used to visualize the network. The replication rate of the reconstructed population genomes was evaluated using the iRep algorithm (45) under default settings based on the genome coverage at each time point obtained by bowtie2 (56) under default settings allowing no mismatches. For the 16S amplicon-based taxonomic units, relative abundance was calculated from the relative abundance of rDNA sequences, and cell-level metabolic activity was estimated using the relative abundances of rRNA sequences normalized against the relative abundance of complementary rDNA sequences (rRNA/rDNA ratio). The relative abundance of reconstructed genomes was calculated as the average coverage of all contigs in a reconstructed genome divided by the total number of reads in the metagenome from which it was assembled. The relative transcriptional activity of a population genome was calculated by summing the TPM (transcripts per million) values of all open reading frames (ORFs) in a genome and dividing by 10^6^, after mapping metatranscriptomic mRNA reads to the reconstructed genomes as described previously (40). The relative production of a taxonomic unit (OTU/ZOTU) or genome was calculated as its relative contribution to total community rRNA or mRNA.

### Data availability.

The amplicon sequencing reads have been deposited in the NCBI Sequence Read Archive under project number PRJNA394936.
