# Supplementary figures and images for: Rare Taxa Exhibit Disproportionate Cell-Level Metabolic Activity in Enriched Anaerobic Digestion Microbial Communities
Source: mSystems. 2019 Jan 22;4(1):e00208-18. doi: 10.1128/mSystems.00208-18 (PMC6343076; doi:10.1128/mSystems.00208-18)

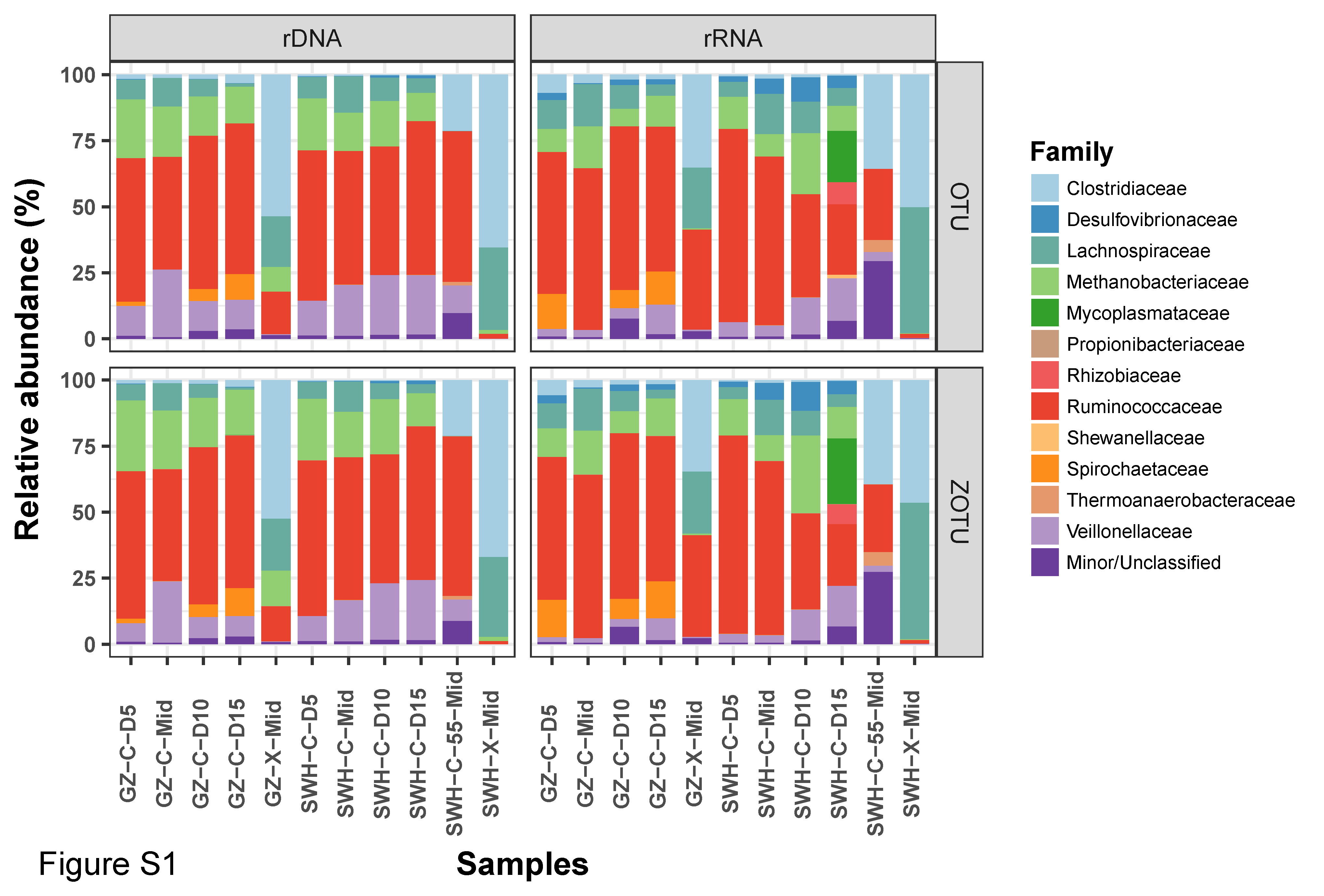

Supplement: FIG S1 [file mSystems.00208-18-sf001.tif]

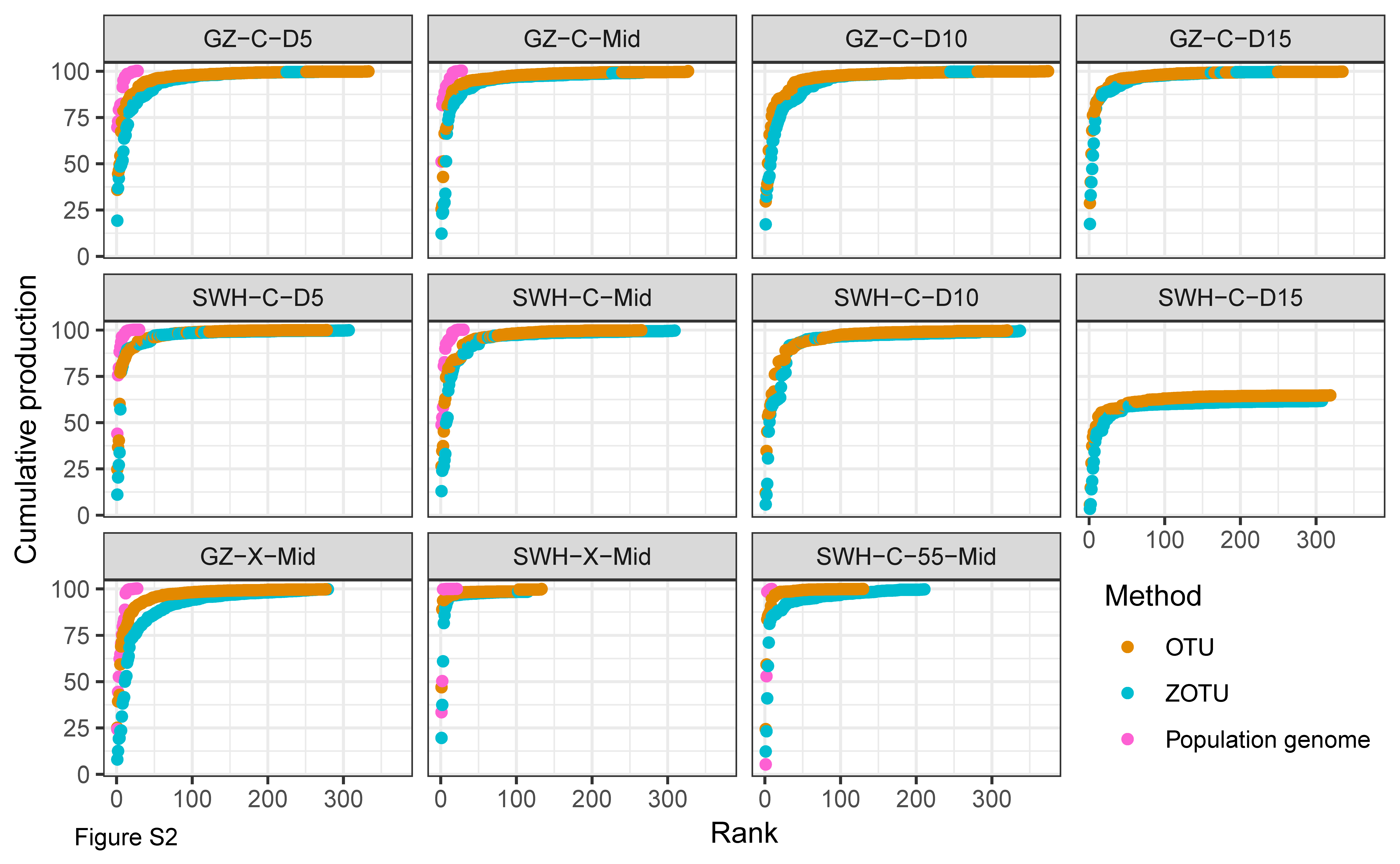

Supplement: FIG S2 [file mSystems.00208-18-sf002.tif]

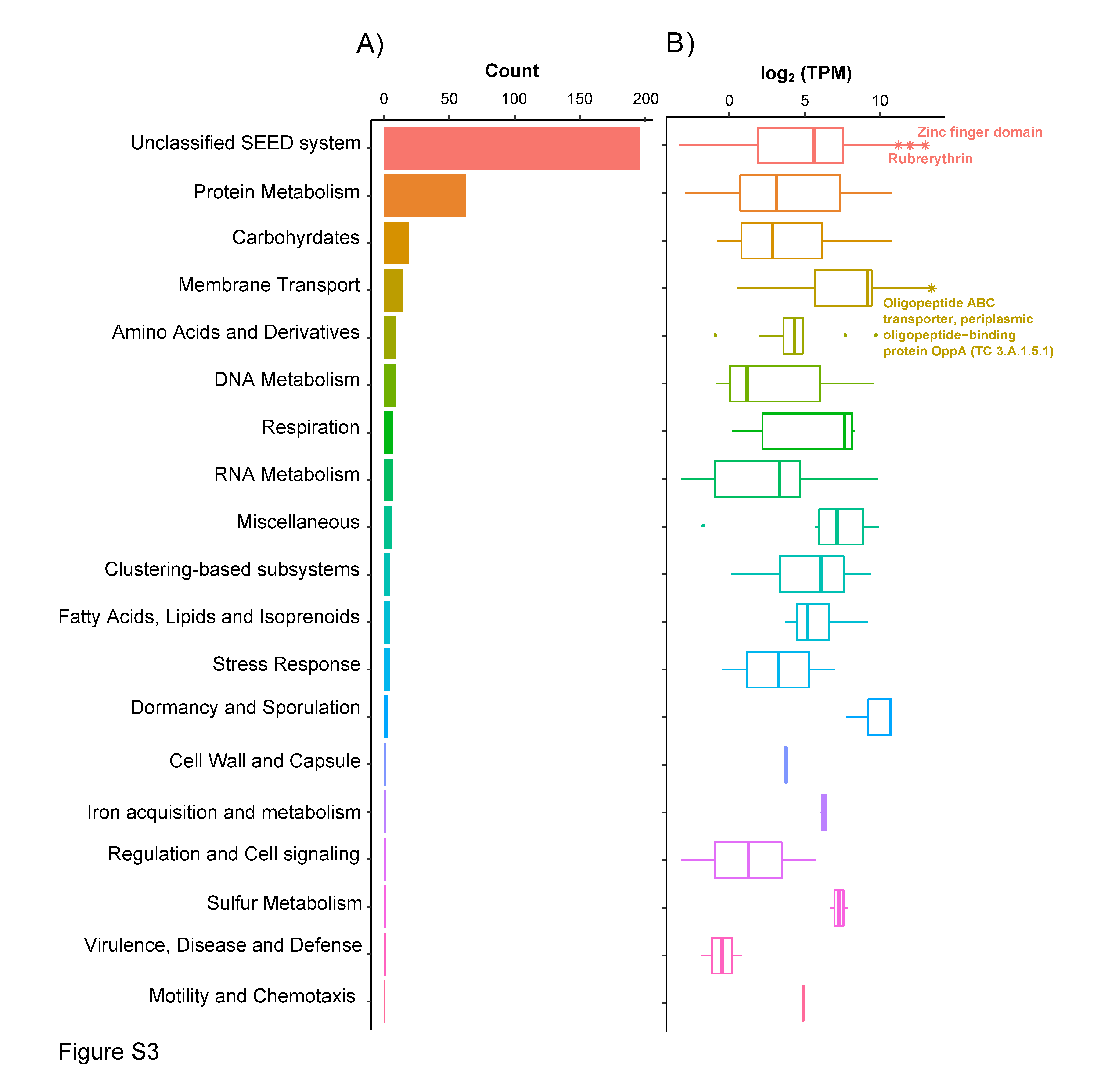

Supplement: FIG S3 [file mSystems.00208-18-sf003.tif]

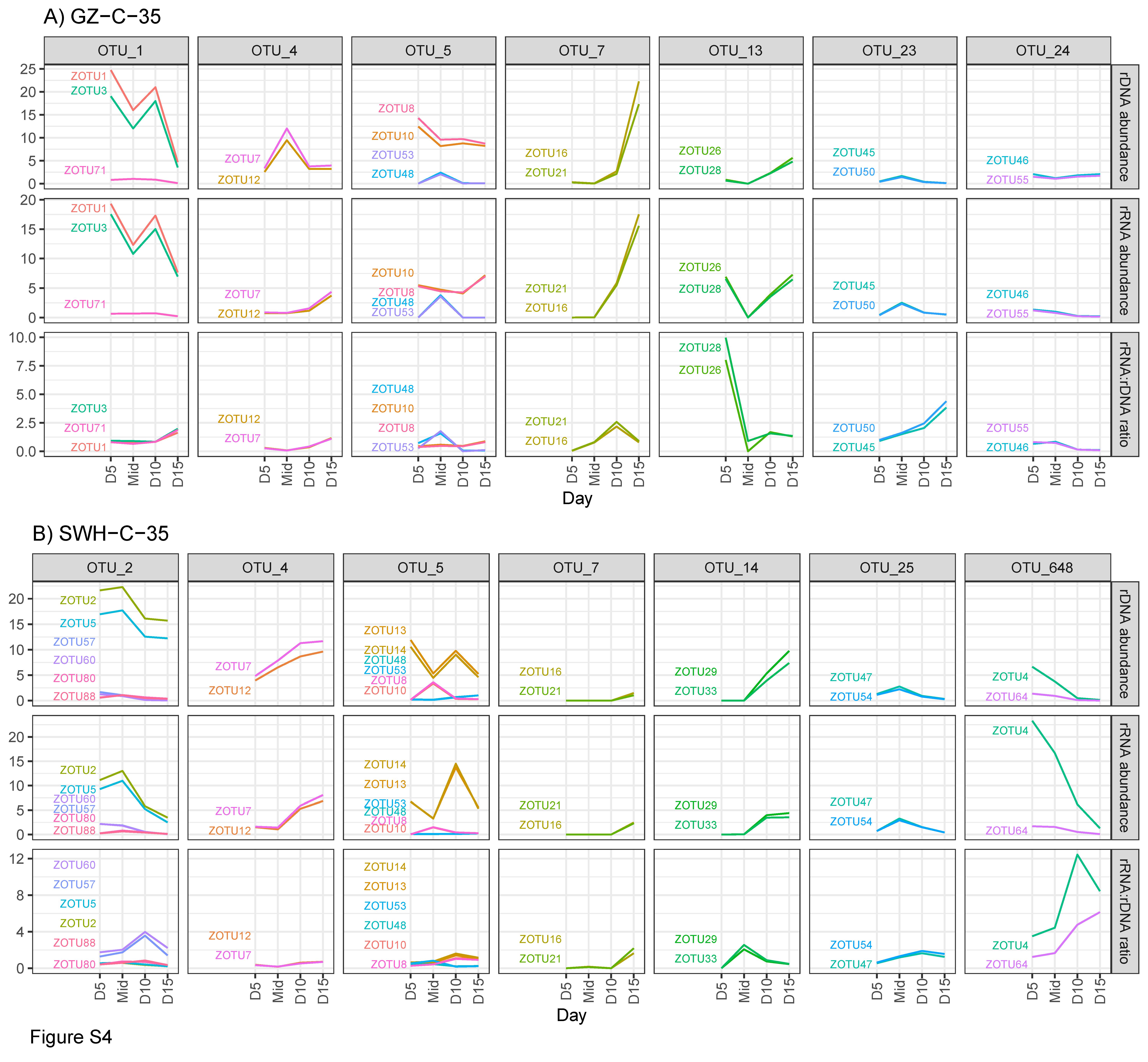

Supplement: FIG S4 [file mSystems.00208-18-sf004.tif]
